# A High Percentage of CD16+ Monocytes Correlates with the Extent of Bone Erosion in Chronic Lymphocytic Leukemia Patients: The Impact of Leukemic B Cells in Monocyte Differentiation and Osteoclast Maturation

**DOI:** 10.3390/cancers14235979

**Published:** 2022-12-03

**Authors:** Paolo Giannoni, Cecilia Marini, Giovanna Cutrona, Katia Todoerti, Antonino Neri, Adalberto Ibatici, Gianmario Sambuceti, Simona Pigozzi, Marco Mora, Manlio Ferrarini, Franco Fais, Daniela de Totero

**Affiliations:** 1 Department of Experimental Medicine, Biology Section, University of Genova, 16132 Genova, Italy; 2 CNR Institute of Bioimages and Molecular Physiology, 20054 Milano, Italy; 3 Nuclear Medicine Unit, IRCCS Ospedale Policlinico San Martino, 16132 Genova, Italy; 4 Molecular Pathology Unit, IRCCS Ospedale Policlinico San Martino, 16132 Genova, Italy; 5 Hematology Unit, Fondazione IRCCS Ca’ Granda, Ospedale Maggiore Policlinico, 20122 Milano, Italy; 6 Department of Pathology, IRCCS Istituto Nazionale dei Tumori G. Venezian, 20133 Milano, Italy; 7 Scientific Directorate, Azienda USL-IRCCS di Reggio Emilia, 42123 Reggio Emilia, Italy; 8 Hematology Unit and Bone Marrow Transplantation, IRCCS Ospedale Policlinico San Martino, 16132 Genova, Italy; 9 Department of Health Sciences, University of Genova, 16132 Genova, Italy; 10 Department of Surgical and Diagnostic Sciences, University of Genova, 16132 Genova, Italy; 11 Pathology Anatomy Unit, IRCCS Ospedale Policlinico San Martino, 16132 Genova, Italy; 12 Department of Experimental Medicine, Anatomy Section, University of Genova, 16132 Genova, Italy

**Keywords:** chronic lymphocytic leukemia, microenvironment, bone remodeling, monocytes

## Abstract

**Simple Summary:**

Bone is a dynamic tissue undergoing resorption and formation. We previously described bone derangement in CLL patients in the advanced stages of the disease and demonstrated the pivotal role of cytokines, released by CLL cells, in stimulating osteoclast differentiation in vitro. Aiming to clarify mechanisms favoring osteoclasto-genesis, we, here, first characterized circulating CLL monocytes and then addressed whether healthy monocytes, under the influence of leukemic cells, acquire phenotypic and functional features distinctive of osteoclast precursors, such as the previously proposed CD16 marker. We determined: (i) a direct correlation between circulating CLL monocytes expressing CD16 and the levels of bone erosion; (ii) that the treatment of healthy monocytes with CLL-conditioned medium up-regulated the expression of CD16, RANK and RANKL and increased the rate of osteoclast formation; (iii) that monocytes polarized toward the M2 phenotype showed higher CD16 expression than M1 and were more prone to differentiate into osteoclasts.

**Abstract:**

Significant skeletal alterations are present in Chronic Lymphocytic Leukemia (CLL) patients; bone erosion, particularly evident in the long bone shaft, appeared increased in the progressive disease stage. Moreover, the partial colonization of the bone with reactive bone marrow we documented via PET-FDG imaging suggests that neoplastic cell overgrowth contributes to bone derangement. Indeed, cytokines released by leukemic B cells impair osteoblast differentiation and enhance osteoclast formation in vitro. CD16, Fcγ-RIIIa, has been previously indicated as a marker of osteoclast precursors. We demonstrate, here, that the percentage of circulating monocytes, CD16+, is significantly higher in CLL patients than in normal controls and directly correlated with the extent of bone erosion. When we assessed if healthy monocytes, treated with a CLL-conditioned medium, modulated RANK, RANKL and CD16, we observed that all these molecules were up-regulated and CD16 to a greater extent. Altogether, these findings suggest that leukemic cells facilitate osteoclast differentiation. Interestingly, the evidence that monocytes, polarized toward the M2 phenotype, were characterized by high CD16 expression and showed a striking propensity to differentiate toward osteoclasts may provide further explanations for the enhanced levels of bone erosion detected, in agreement with the high number of immunosuppressive-M2 cells present in these patients.

## 1. Introduction

Chronic Lymphocytic Leukemia (CLL) is characterized by the clonal expansion of leukemic B cells, which progressively accumulate in peripheral blood, lymph nodes and bone marrow (BM). Interactions between CLL cells and accessory cells of the microenvironment, as well as the dysregulation of the immune system control, contribute to increasing leukemic cells survival [1,2], further causing phenotypical and functional modifications in the interacting cell types [3]. We recently demonstrated that CLL cells inhibited osteoblast differentiation and stimulated osteoclast formation; osteoclasts, in turn, enhanced CLL cell viability and proliferation [4], thus, creating a reciprocal self-sustaining loop. The observation that patients with an advanced disease stage (Binet C versus A) [5] have higher levels of bone erosion at the long bone shaft further suggests that the progressive expansion of leukemic B cells affects physiological remodeling of the bone tissue.

Osteoclast precursors (OCPs) are represented by myeloid-lineage cells of the BM, which enter in the circulation, migrate toward the bone and here differentiate into mature osteoclasts [6,7,8]. Studies with PBL from human subjects have demonstrated that OCPs are encompassed in the CD14+ circulating population. Interestingly, it has been shown that monocytes with higher CD16 expression exhibit an increase in their osteoclastogenic potential [9,10]. CD16, the Fc gamma type III low-affinity receptor for IgG, is a RANK co-stimulator and, together with DAP12, drives calcium oscillation necessary for NFATc1 production, a key transcription factor in osteoclast differentiation. Phosphorylation of CD16- and of DAP12-ITAM domains primes SYK tyrosine, leading, in turn, to BLNK/SLP76 activation and recruiting TEC kinase and phospholipase C gamma2 to form the osteoclastogenic signaling complex, crucial for activation of calcium signaling. Studies on BTK/TEC doubly deficient mice, that develop an osteopetrotic phenotype, have, indeed, demonstrated a pivotal role for these kinases in coupling signals from this complex with those provided by RANK/ RANKL activation [11,12,13].

Circulating monocytes have been divided into three subsets, based on the levels of CD16 expression: “classical” (CD14++CD16−), “intermediate” (CD14++CD16+) and the “non-classical” (CD14+CD16++) monocytes. The expression of CD16 in monocytes, rare in normal conditions, is two- to four-fold increased during infections and inflammation [14,15,16,17]. CD14+CD16+ monocytes also appear expanded in many diseases characterized by bone derangement, such as Gaucher disease or psoriatic arthritis [18]. Moreover, the number of “non-classical monocytes” is significantly higher in the BM of patients with Multiple Myeloma (MM) than in healthy controls [19] and is directly correlated with the percentage of malignant plasma cells. Interestingly, CD14+CD16+ cells, sorted from the BM of MM patients and cultured ex vivo, were more pro-osteoclastogenic than the CD16-negative counterpart [20]. The increase in the percentage of CD14+CD16+ monocytes appears also to be associated with a higher incidence of bone fractures in childhood obesity [21]. Collectively, these observations prompted us to evaluate, in a cohort of PBMCs obtained from 35 CLL patients, the percentages of the three different subsets of monocytes, in comparison with PBMCs from monoclonal B-cell lymphocytosis (MBL) patients or from normal donors, trying to determine potential correlations between the expansion of a specific subset and the entity of bone erosion. From an experimental point of view, we also investigated whether conditioned media (CLL-cm), derived from CLL B cell cultures, could modulate the expression of molecules involved in osteoclast differentiation, such as CD16, RANK and RANKL.

## 2. Materials and Methods

### 2.1. Patients

Thirty-seven patients were enrolled in the present study (see Supplementary Table S1), including subjects already examined in a previous work [5]. Peripheral blood and sera were collected after informed consent and the study was approved by the Ethics Committee of IRCCS Policlinic San Martino Hospital. The THP-1 monocytic cell line (ICLC Ospedale Policlinico San Martino, Genova, Italy) was also used in selected experiments.

### 2.2. Determination of the Percentage of CD16-, RANK- and RANKL-Positive Monocytes from Peripheral Blood of CLL or of MBL Patients or of Healthy Donors

The rate of the expression of CD16 (Fc gamma RIII), RANK and RANKL molecules was evaluated by flow cytometry analysis in circulating monocytes from the peripheral blood of CLL and MBL patients and compared with levels of expression in monocytes present in PBMCs from healthy donors. PBMCs obtained after Ficoll gradient separation (Euroclone S.p.A. Milan Italy) were double stained with anti-human CD14 fluorescein-conjugated (FITC) and CD16 phycoeritrin-conjugated (PE) moAbs (Immunotools, Friesoythe, Germany) for 30 min at 4 °C in the dark. After this incubation time the samples were washed twice, resuspended in PBS + FCS 2% (Euroclone) and immediately analyzed via the use of an FACSCanto (BD Biosciences/Pharmingen, San Jose, CA, USA). The percentage of monocytes positive for RANK and RANKL was also examined by three color staining tests with CD14-FITC or CD16-FITC (Immunotools), RANK-PE (Biotechne-R&D System, Milan, Italy) and RANKL-Allophycocyanin (APC)-conjugated moAbs (Biolegend, Amsterdam, The Netherlands). Monocytes were identified and gated based on physical parameters (FSC versus SSC) and on CD14+ positivity. FMO (fluorescence minus one) control was considered the sample stained with CD14-FITC only and was performed for all analyzed specimens. At least 50,000–100,000 events were acquired for each sample. The comparison of the percentages of monocytes belonging to the three different groups (non-classical, intermediate and classical) among CLL cases and MBL cases or healthy donors was evaluated normalizing to 100% the percentage of CD14+ monocytes.

### 2.3. Evaluation of Trabecular and Compact Bone Volume (IBV and CBV) and Correlation with CD16 Expression

All assessed patients were subjected to whole-body X-ray CT scan using a 16-detector row helical scanner (Biograph Siemens, Erlangen, Germany). Gantry rotation speed was set at 0.5 seconds using a table speed of 24 mm per rotation. Image analysis was performed according to our previously validated procedure that exploits the different attenuation coefficient to quantify compact (CBV) and trabecular bone volume (IBV) in all trans-axial CT images. The ratio between trabecular and total skeletal volume (IBV/SV, where SV is the sum of IBV and CBV) was used to estimate the erosion of the whole skeleton of axial segments (vertebral bodies and sternum) and appendicular long bone shafts. All volumetric data were normalized according to patient ideal body weight (IBW), estimated according to the Robinson equation [22]. Correlation between percentage of CD14+/CD16+ cells and IBV/SV ratio (as an index of bone erosion) was tested by linear regression analysis using the least-squares method according to the routine of SPSS software (SPSS; version 20.0). The percentages of monocytes CD14+CD16++ (non-classical); CD14++CD16+ (intermediate); and CD14++CD16− (classical) referred to 100% of CD14+ monocytes.

### 2.4. Determination of the Expression of CD16, RANK and RANKL on Healthy Monocytes following Their Cultures with Conditioned Media or Sera from CLL B Cells by Cyto-Fluorographic Analysis

The expression of CD16, RANK or RANKL molecules was evaluated on purified monocytes obtained from healthy donors, before and after treatment with CLL-conditioned media (CLL-cm) derived from various patients. Monocytes were purified from PBMCS of healthy donors by the use of anti-CD14 microbeads (Miltenyi Biotech GMbH Friederich-Ebert, Germany), as previously described [4], and were cultured in 24-well plates with basal medium only (RPMI 1640 +3% FCS) (Lonza, Walkersville, MD, USA) or with the addition of CLL-conditioned media (CLL-cm: 300 µL) for 48 h or for different times as indicated. CLL-cm were derived from B CLL cell culture as previously described [4]. Cells were then detached from each well by gentle scraping, double-stained with the anti- human CD14-FITC and CD16-PE moAbs (Immunotools) and incubated at 4 °C for 30 min in the dark. To determine RANK and RANKL expression we further performed three-color staining with the anti-human CD14-FITC (Immunotools), -RANK-PE (R&D System) and -RANKL-APC (Biolegend) moAbs. Cells were then washed twice, resuspended in PBS+ FCS 2% and analyzed by flow cytometry using an FACS/Canto (BD Biosciences/Pharmigen). In selected experiments the expression of CD16 was studied after stimulation with MCSF (25 ng/mL) + RANKL (25 ng/mL) (Miltenyi) for a longer time (15–21 days): in these experiments double staining was performed with the anti-human- CD14-FITC and -CD16-PE moAbs in two distinct populations of cells clearly different for size and present in each well. One population, which we called UP-POP, appeared smaller and floating and was, thus, detached by only washing, while the other population, larger and firmly attached to the plastic, named DOWN-POP, was detached by both washing and intensive scraping. In a different set of experiments we further addressed CD16 expression in monocytes treated with recombinant human (rh) IL-10 (20 ng/mL) or with rh-TGFβ (20 ng/mL) (Milteny) for 48 h. The expression of CD14 and CD16 was also evaluated by immunofluorescence in monocytes seeded in 8-well chamber slides (Corning, New York, NY, USA) and differentiated toward osteoclasts with the classical MCSF + RANKL stimulation. Briefly, purified monocytes were first seeded in chamber slides in a total volume of 400 μL of RPMI + FCS 10%. MCSF (25 ng/mL) + RANKL (25 ng/mL) were added one day later and after one week. After 15 days of culture the cells were first fixed with PFA 4% in PBS for 15 min. After three washes with PBS +FCS 2%, the cells were incubated for 30 min in the dark with the anti-human CD14-PE- and CD16-FITC moAbs (Immunotools). Cell nuclei were, therefore, identified by standard staining with 4’,6-diamidin-2-fenilyndole (DAPI, Invitrogen-ThermoFisher Scientific, Milan, Italy) and images were taken by the use of an Olympus BX5 1 fluorescence microscope.

### 2.5. Evaluation of Proliferation and Migration of Healthy Monocytes in Response to Conditioned Media from CLL B Cells

The evaluation of proliferation of monocytes in response to CLL-cm was performed by staining monocytes previously purified by anti-CD14 microbeads (Miltenyi) with carboxylfluorescein diacetate succinimidyl-ester (CFSE; Invitrogen-ThermoFisher Scientific). Briefly 3 × 10^6^ of purified monocytes from healthy donors was first washed with PBS and further stained with CFSE at 37 °C for 8′. After two washes cells were resuspended in RPMI + FCS10%, dispensed at a concentration of 1 × 10^5^ monocytes/well in a 24-well plate and further cultured with only medium (RPMI + FCS10%) or with the addition of conditioned media from CLL B cells cultures (300 μL in 1ml final volume of RPMI + FCS10%). After 48 h, 72 h and 6 days monocytes were harvested by gentle scraping with a rubber policeman and CFSE fluorescence was acquired by flow cytometry (FACSCANTO, Beckton-Dickinson). The presence of multiple peaks identifying multiple divisions of cells gradually losing CFSE fluorescence intensity was further analyzed by the use of the FlowLogic ^TM^ software (Inivai Technologies, Mentone, Victoria 3194, Australia). A logical gate was designed on the large and activated cells. Migration of purified healthy monocytes was evaluated in response to FCS 10% or to CLL-cm or two stromal cell lines, HS5 (a Kind gift from Prof. S. Ferrini, IRCCS Ospedale Policlinico San Martino, Genova, Italy) and Fibrogem (in-house derived cell line). To this aim 500 μL of RPMI + FCS 10% or 300 μL of CLL-cm or of HS5-cm or of Fibrogem-cm, +200 μL of RPMI, were first dispensed in the bottom of each well of a 24-well plate. Thus, 2 × 10^5^ monocytes in a final volume of 500 μL of RPMI were further dispensed upside each trans-well (Corning). After 4 h of incubation, 100 μL from the bottom of each well was transferred in a vial and counted for 1 min to quantify the number of events relating to the number of migrated cells. At least three samples were determined for each well.

### 2.6. Generation of Osteoclasts and Determination of the Number of Osteoclasts after TRAP Staining

Monocytes (1 × 10^5^) purified from healthy PBMCs by conjugated anti-CD14 microbeads (Miltenyi) were seeded in 24-well plates in medium (RPMI1640 + FCS10%) and MCSF (25 ng/mL; Miltenyi) and RANKL (25 ng/mL; Miltenyi) were added one day after. In selected experiments monocytes were pre-activated with CLL-cm (300 μL/mL), TGFβ (20 ng/mL) or IL-10 (20 ng/mL) for 48 h, before the addition of MCSF (25 ng/mL) and RANKL (25 ng/mL). The neutralizing anti-TGFβ antibody (400 ng/mL: R&D System, Minneapolis, MN, USA) and the purified anti-CD16 antibody (13µg/mL, anti—Hu/NHP CD16, clone 3G8, ThermoFisher eBioscience ^TM^, ThermoFisher Scientific Italy, Rodano, Milano, Italy) was utilized in some experiments and added contemporarily to CLL-cm, to determine potential inhibition of osteoclast formation. In another set of experiments monocytes were pre-treated with supernatants derived from THP-1 cells previously polarized toward M1, M2a or M2c. Polarization of THP1 cells was performed by stimulating 500,000 cells/well (6-well plate) with PMA (20 ng/mL) for 24h and then adding LPS (250 ng/mL) + IFNγ (20 ng/mL) (M1), or IL4 (20 ng/mL) (M2a) or IL10 (20 ng/mL) (M2c) for 48 h more. Supernatants from THP1-M1-, -M2a- or -M2c-induced cells were, therefore, collected, filtered and frozen until use. Monocytes from PBMCs of healthy donors, first purified by CD14 microbeads (Miltenyi) were also directly polarized by previous stimulation with PMA (150,000 cells/mL in 24-well plates) and subsequent activation with the different growth factors, as described above, to induce M1, M2a M2c cells. Polarized monocytes were then stimulated with MCSF (25 ng/mL) and RANKL (25 ng/mL). After ten days, the number of osteoclasts was determined by counting Tartrate-Resistant Alkaline Phosphatase-positive (TRAP+) cells on an inverted Olympus CKX-41 microscope. Triplicate samples were prepared for each culture condition and at least six image fields were taken for each well. TRAP+ cells showing at least three nuclei were considered as osteoclasts.

### 2.7. TRAP Staining and Bone Resorption Assay

Osteogenically induced monocytes were washed in PBS and fixed in a fixation solution (4% PFA in acetate/citrate-buffered solution, pH 6.0) for 15 min at RT. Cells were then stained according to the protocol provided with the Tartrate-resistant acid phosphatase detection kit (Sigma-Aldrich, kit. N. 387A, Milan, Italy), by exposing fixed cells to a freshly prepared tartrate-containing solution for 60 min at 37 °C; the staining solution was then removed and cells were washed in distilled water and photographed. At least three different cell cultures were assessed for TRAP staining, in different experimental conditions. At least 3 images were taken for each well/experimental condition/cell culture. For each image the area of frankly trinucleated TRAP+ cells was measured by using the public software NIH Image J (version 1.48v; http://imagej.nih.gov/ij; accessed on 3 October 2012) tools. Cells were categorized as small or large, according to a cut-off area of 25,000 pixels.

CD14+ purified monocytes from healthy donors were seeded in 24-well plates coated with an inorganic crystalline material (Corning, Osteo Assay Surface multiwell plates; cat.n. CLS3987-4EA) and induced toward osteoclast differentiation, with/without previous polarization, as described above. After 14 days the cells were stripped from the plates by treatment with a 10% sodium hypoclorite solution, for 10 min; wells were thoroughly washed and dried at RT for 4 h. Images of triplicate experimental conditions of each culture were acquired and the areas of the resorption pit (expressed in pixels) were determined using the public software NIH Image J. The bone resorption activity in the different experimental conditions was expressed as the ratio of the resorbed pits vs. the total image field area.

### 2.8. Determination of IL-10 and TGFβ Levels in CLL-Conditioned Media

The levels of IL-10 and TGFβ in conditioned media from CLL-cultured cells were determined via a quantitative Elisa assay (Millipore Milliplex Kit) (Burlington, MA, USA).

### 2.9. Immuno-Histochemical Analyses of Bone Biopsies from CLL Patients

Tissue was fixed in B5 (Sigma Aldrich) for 2.5 h and decalcified for 3 h before processing. B5-fixed, paraffin-embedded blocks were sectioned at 2 mm, deparaffinized and rehydrated. Endogenous peroxidase was blocked with 5% H2O2 for 10 min. Immunoreactions for TRAP staining (clone 26E5 Life Technologies, Monza, Italy) and CD16 staining (NCL-L-CD16; Leica Biosystems, Buccinasco, Milan, Italy) were performed using the automated BenchMark Ultra XT immunostainer (Ventana Medical Systems, Oro Valley, AZ, USA). Standard heat-based antigen retrieval (EDTA-borate buffer: pH 8.0) was performed for TRAP and for CD16 (64 min and 40 min, respectively). The Ultraview DAB (for TRAP) and the ultraview RED (for CD16) detection Kits (Ventana Medical System) were used to stain serial sections (5 µm). Slides were then processed for standard mounting. Images acquired from each section were scored for TRAP+ and/or CD16+ cells.

### 2.10. In Silico Interrogation of Public Data on Human Monocytes from Healthy Donors or from CLL Patients

Expression levels of selected genes (FCG3, NFATC-1, RANKL, RANK) were extracted and analyzed from public databases. Robust Multiarray Average expression levels [23] from GeneChip^®^ Human Genome U133A and B arrays (HG-U133; Affymetrix) were obtained for M1- and for M2-activated macrophage biological triplicates, respectively (GSE5099 [24]). Expression levels [25] of matched CD16+ and CD16- peripheral blood monocytes, isolated from 4 different healthy donors, were analyzed on the GeneChip^®^ HG-U133 Plus 2.0 Array (Affymetrix) (GSE16836 [26]). The three monocyte subsets, the CD14++CD16− classical, the CD14++CD16+ intermediate and CD14+CD16+ non-classical, obtained from 4 individual donors in duplicates, were analyzed on the Illumina human-6 v2.0 expression beadchip (GSE25913, [27]). When multiple probes were used to assess the expression of target gene(s) the mean result of all probes was considered for each experiment.

### 2.11. Statistics

ANOVA and Tukey’s post hoc test were used. Two-sided Student’s *t*-test or linear regression analysis were also used. The levels of statistical significance are indicated as *, ** or *** for *p* ≤ 0.05, *p* ≤ 0.01 or *p* ≤ 0.001, respectively.

## 3. Results

### 3.1. The Percentage of Circulating Monocytes Expressing CD16 Is Significantly Higher in CLL Than in MBL Patients or in Healthy Donors

**Figure 1:**
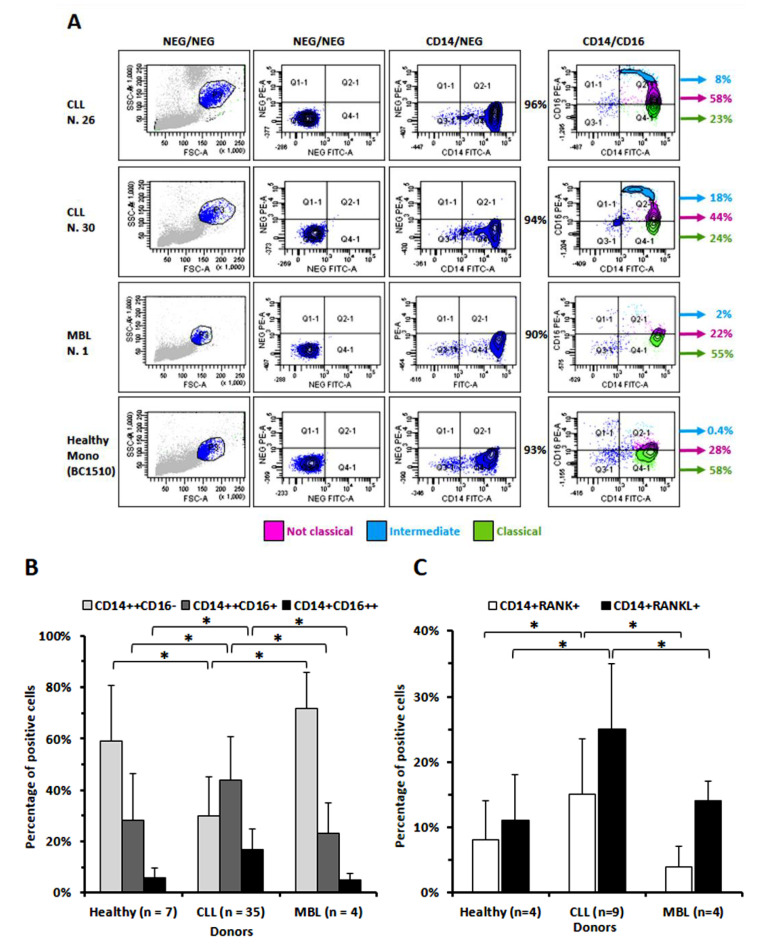
Cyto-fluorographic determination of the percentage of circulating “classical”, “intermediate” and “non-classical “monocytes, and assessment of monocytes expressing RANK and RANKL in a panel of CLL patients as compared with MBL patients or with healthy donors. (**A**) Dot plots display percentages of the three different monocyte subtypes (classical, intermediate and non-classical) in representative subjects of each group of donors examined. The three populations are here represented by different colors: light blue for non-classical, pink for intermediate and green for classical monocytes, as identified by 3 different gates designed on the basis of the CD16 expression levels. FMO (fluorescence minus one) control is the sample stained with CD14-FITC only that was performed in all the samples analyzed. (**B**) Histograms highlight that in CLL patients there was a relevant reduction in “classical” monocytes and a contemporary significant increase in the “intermediate” and “non-classical” subsets, with respect to MBL and healthy donors. (**C**) Monocytes from CLL patients showed a trend of increased positivity for both RANK and RANKL expression in comparison with healthy or MBL donors. Histograms are mean ± SD of the indicated n cases as evaluated in cyto-fluorographic analyses. Statistical significance was determined by using ANOVA and Tukey’s post hoc test.

**Figure 2:**
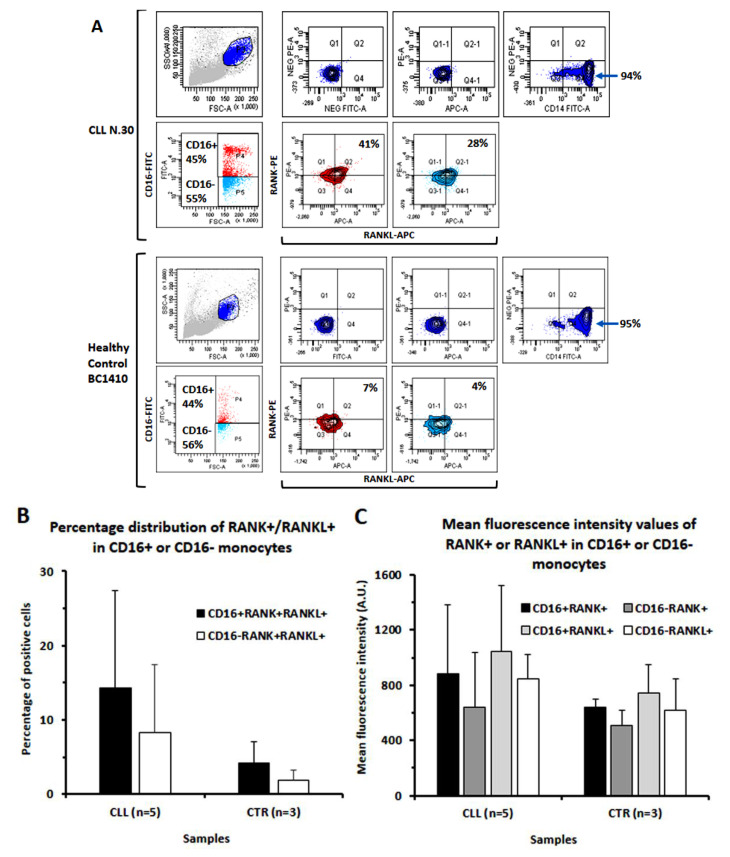
Cyto-fluorographic determination of the percentage of CD16-positive or CD16-negative monocytes contemporarily expressing RANK and RANKL. (**A**) Dot plots display percentages of CD16+/RANK+/RANKL+ (red population) or CD16−/RANK+/RANKL+ (light-blue population) monocytes of a representative CLL case (N.30) as compared to monocytes from a healthy donor (BC1410). Two separate gates (p4 for CD16+ and p5 for CD16−) were applied to differentiate positive and negative cells based on FSC-A and CD16-FITC parameters. (**B**) Histograms highlight that CD16+ monocytes tend to co-express RANK and RANKL and that the triple-positive cells appear in larger proportion in CLL cases (n = 5 examined cases) than in controls (n = 3). (**C**) The same tendency is also shown in terms of mean fluorescence intensity (m.f.i.) within CD16-positive or -negative monocytes, in CLL or in healthy controls.

We first compared the percentage of circulating monocytes expressing CD16 in CLL patients with that present in Monoclonal B Lymphocytosis (MBL) patients or in healthy donors. We, therefore, evaluated the percentages of the three sub-populations of monocytes distinct on the bases of CD14 and CD16 expression: the “classical monocytes”, CD14++ CD16-, representative of the largest population found in normal controls, the “intermediate monocytes”, CD14++CD16+ or the more rare “non-classical monocytes” CD14+ CD16++. As shown in Figure 1A,B, the percentages of “intermediate” and of “non-classical monocytes” were significantly increased in CLL patients (n = 35) as compared to MBL (n = 4) or healthy donors (n = 7), while the percentage of “classical monocytes” was significantly reduced. Higher levels of RANK and RANKL expression were also detectable in monocytes from CLL patients than in those from MBL patients or from healthy donors (Figure 1C). Furthermore, RANK and RANKL appeared more expressed in CD16+ monocytes when a triple determination was performed. Moreover, the percentage of triple-positive monocytes was higher in monocytes from CLL cases than from healthy donors (Figure 2A–C).

### 3.2. In CLL Patients, the Percentage of CD16+ Monocytes Directly Correlated with the Levels of Bone Erosion

**Figure 3:**
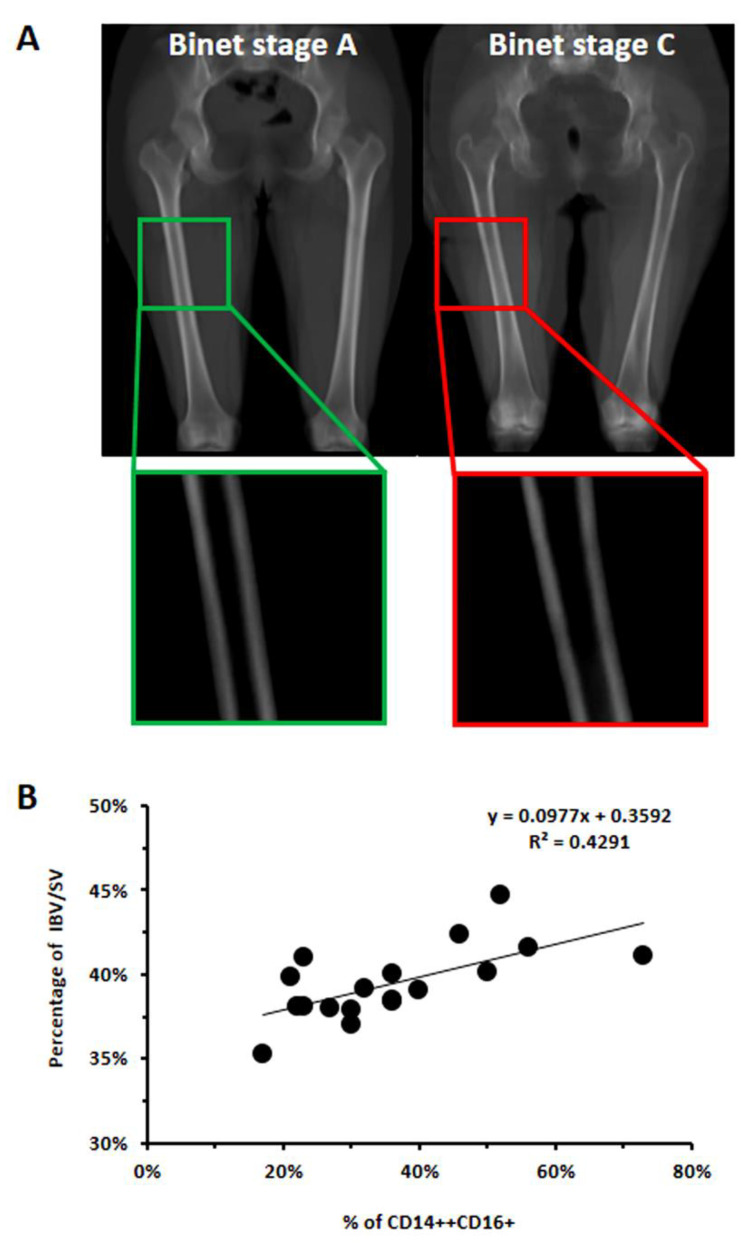
Representative radiograms of long bone shaft (femurs) in Binet stage A and C patients and correlation analysis of bone erosion levels and circulating CD14+CD16+ monocytes. (**A**) Enlarged X-ray scans document the thinning of the cortical bone and enlargement of the intra-bone volume of the femur diaphysis in a representative Binet C patient as compared to a Binet stage A one, among those included in this study. (**B**) The presented plot evidences a direct correlation between the percentage of circulating CD14++CD16+ (intermediate) monocytes and the index of bone erosion (IBV/SV ratio).

As we previously reported, CLL patients show a significant erosion of compact bone that is paralleled by an expansion of intra-bone volume with respect to control subjects (39 ± 2 vs. 32 ± 3%, *p* < 0.001). The degree of bone erosion is, furthermore, evident in progressive disease stages [5,28], as also evidenced in Figure 3A. We then evaluated if there was a correlation between the percentage of the three monocyte subpopulations and whole-body bone erosion (IBV/SV) in a subset of 17 CLL patients in different stages of disease (six subjects of Binet stage A, six of stage B and six of stage C). As shown in Figure 3B, we could determine a significant correlation between the entity of bone erosion and the percentage of monocytes classified as” intermediate” CD14++16+ (*r* = 0.42).

### 3.3. CLL-Conditioned Media Derived from Cultures of CLL B Cells Up-Regulated CD16 on Healthy Monocytes

**Figure 4:**
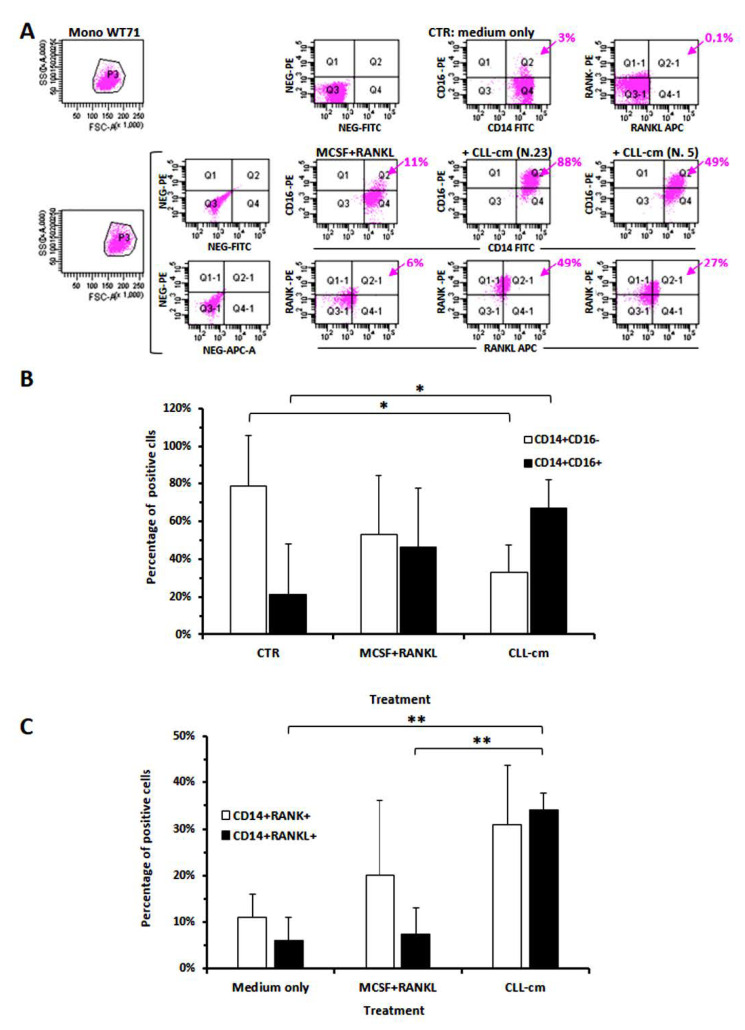
Modulation of CD16, RANK and RANKL expression in CD14+ healthy monocytes by CLL-cm treatment. (**A**) Presented dot plots show that CD16 expression was markedly induced when healthy monocytes (WT71) were cultured with CLL-cm (from two representative cases) for 48 h. (**B**) CD16 is significantly increased in purified monocytes treated with CLL-cm derived from B-cell cultures of 7 CLL patients. Histograms represent the means of 3 experiments ±SD using purified monocytes from 3 healthy donors. (**C**) The addition of CLL-cm also increased the percentage of CD14+ monocytes expressing RANK and RANKL, as evaluated in purified monocytes derived from 3 healthy donors cultured with CLL-cm (n = 6) for 48 h. Histograms are mean ± SD of three experiments. Statistical significance was determined by using ANOVA and Tukey’s post hoc test.

It is well known that RANKL represents the master cytokine involved in osteoclast differentiation. However, other co-receptors may cooperate with RANK to amplify the monocyte response and promote their maturation. The low-affinity receptor FcγRIIIa, or CD16, may facilitate actin cytoskeleton re-organization and may contribute in regulating multiple-cell fusion together with other RANK co-receptors [29,30]. Since we previously determined enhanced osteoclast formation from monocytes cultured with media derived from CLL B cell cultures (CLL-cm) [4], we evaluated here whether healthy monocytes, cultured with CLL-cm, up-regulated the expression of CD16, RANK and RANKL. As shown in Figure 4A,B, the addition of CLL-cm significantly increased CD16 expression in monocytes derived from healthy donors. CLL-cm also enhanced the expression of RANK and of RANKL (Figure 4C). These findings may collectively suggest that cytokines, released by leukemic cells, stimulate the up-regulation of CD16, of RANK and of RANKL, possibly contributing to monocyte differentiation toward an osteoclast precursor stage.

### 3.4. Evaluation of CD16 Expression along Osteoclast Maturation in Healthy Monocytes

**Figure 5:**
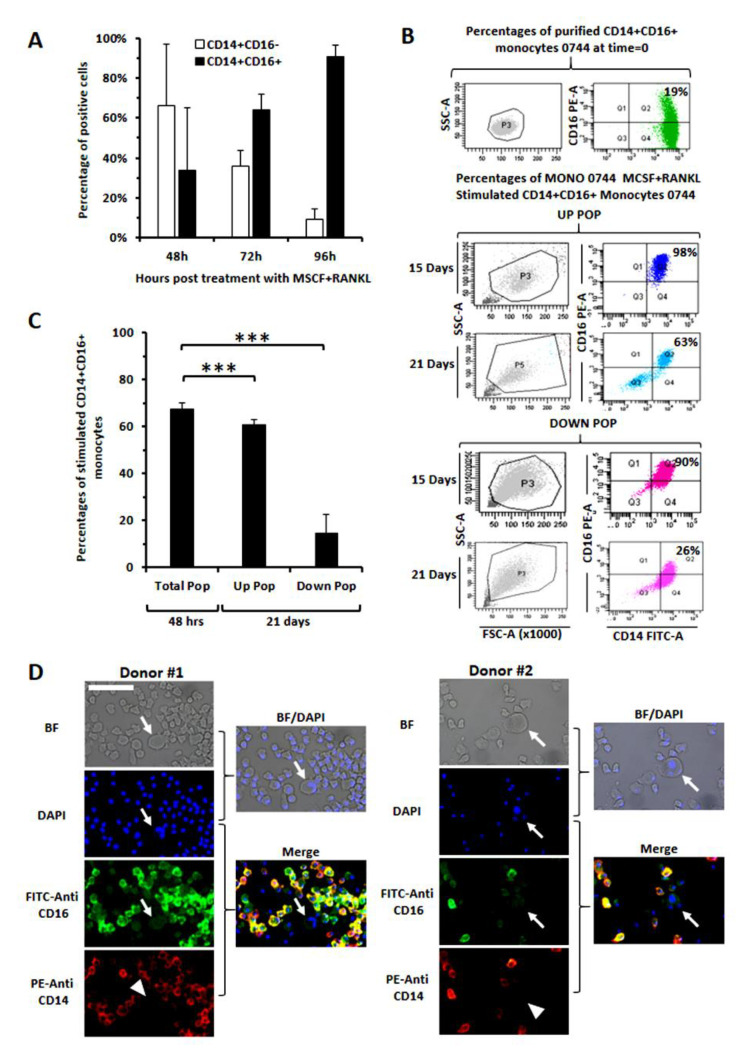
Evaluation of up-regulation and preservation of CD16 during transitional stages of monocyte differentiation toward osteoclasts. (**A**) Factors promoting osteoclast differentiation in vitro (MCSF + RANKL) strongly increased the percentage of CD16+ monocytes within 72–96 h of exposure. Data are means of monocytes derived from three different donors. (**B**) CD16 was highly expressed in both UP (smaller) and DOWN (larger) populations after 15 days of stimulation and resulted instead in significantly decreased only in the DOWN population at later times. (**C**) Analysis of CD16 expression in UP and DOWN population as evaluated in monocytes from three different donors. (**D**) Images display strong CD16 positivity in a large number of small monocytes and a weaker expression in osteoclasts (frankly tri-nucleated as indicated by arrows) which also have down-regulated CD14 (arrow-heads). Images are representative of monocytes from 2 healthy donors studied after 15 days of classical stimulation with MCSF + RANKL. All images were acquired at the same enlargements (bar: 6 µm).

CD16 antigen has been proposed as a marker of osteoclast precursors in psoriatic arthritis or in Multiple Myeloma: CD14+CD16+ cells were, in fact, more prone to differentiate toward osteoclasts, at least in these two diseases [9,20]. To gain more insight on the role of CD16 in osteoclast differentiation, we, here, addressed if CD16 could be gradually acquired by monocytes during the transitional stage, leading to their maturation. We observed a significant CD16 up-regulation after the classical in vitro stimulation with osteoclast-promoting factors (MCSF + RANKL): the percentage of CD14+CD16+ cells, low at basal conditions, appeared strongly enhanced after 48–96 h of activation (Figure 5A). Trying to clarify whether CD16 was maintained or lost in mature osteoclasts, we further decided to analyze the expression of CD16 at different time points after MCSF + RANKL stimulation of healthy donor monocytes. The observation, via an inverted light microscope, of the activated and cultured cells in a 24-well plate, however, revealed the presence of two populations, clearly distinct for size and adhesiveness. We, therefore, performed CD16 staining separately by recovering the first population (smaller cells, less adherent to the plastic, named UP-POP) by multiple washes and by scraping and intensively washing the second population (enlarged cells strongly adherent to the plastic, named DOWN-POP). As shown in Figure 5B, after 15 days of culture, CD16 appeared expressed at high levels on both the UP- and DOWN-POP, while, after 21 days, it was significantly down-regulated in the DOWN-POP cells only. Collectively, the CD16 variation in UP- and DOWN-POP was assessed after 21 days in monocytes purified from three different healthy donors, further confirming a decrease in CD16, massively evident in the DOWN-POP (Figure 5C). In addition, through an immunocytochemistry approach, we here show that a discrete percentage of monocytes, derived from two other healthy donors, co-express CD14 and CD16, while the tri-nucleated cells—osteoclasts by definition— exhibit a weak CD16 expression and have lost CD14 (Figure 5D). Altogether, these findings may suggest that the smaller cells represent osteoclast precursors, while the larger ones may be representative of more differentiated elements. Proof of CD16 up-regulation in putative precursors, as well as its down-regulation in more mature osteoclasts, appears consistent with a theoretical role of this molecule in the process of amplification and differentiation at a relatively early stage, possibly facilitating the fusion of multiple progenitors.

**Figure 6:**
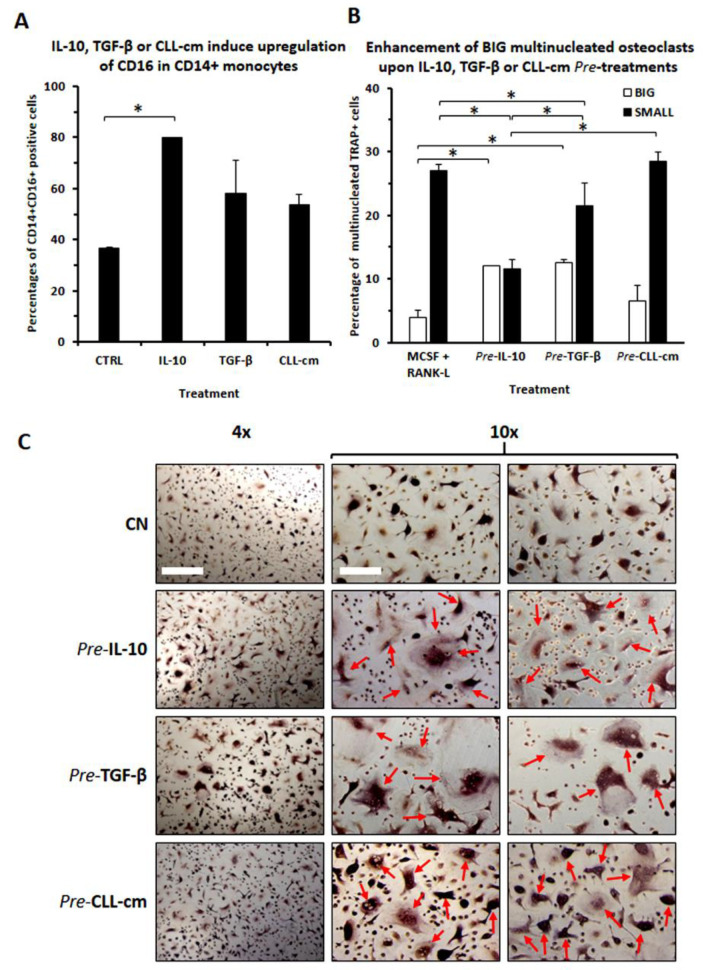
Effects of cytokines exposure on CD16 expression and on osteoclast differentiation in healthy monocytes. (**A**) Monocytes pre-stimulated with cytokines potentially released within the microenvironment, such as IL-10 and TGFβ, or with CLL-cm, showed up-regulation of CD16 expression after 48 h of treatment, as determined by cyto-fluorographic analysis. (**B**) CD16 up-regulation, due to cytokine pre-treatment, appeared further paralleled by an increase in multinucleated BIG osteoclast formation. Histograms depict the mean ± SD of monocytes treated with CLL-cm from 3 CLL cases repeated in 2 separate experiments. (**C**) Monocytes pre-stimulated with IL-10, TGFβ and CLL-cm gave rise to a higher number of frankly multinucleated TRAP+ osteoclasts (arrows) as compared to MCSF + RANKL treatment only (CN), as depicted in images from a representative experiment. Bars correspond to 60 and 25 µm for 4× and 10× images, respectively. Statistical significance was determined by using ANOVA and Tukey’s post hoc test.

**Figure 7:**
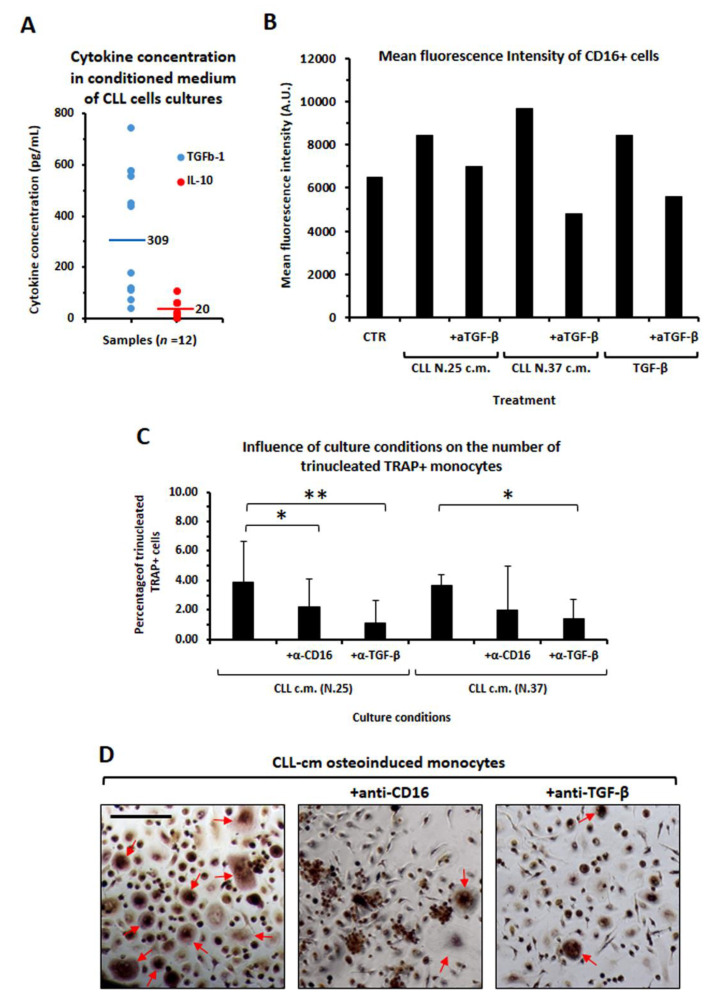
Determination of IL-10 and TGFβ concentration in CLL-conditioned media and evaluation of the osteoclastogenesis inhibition by anti-TGFβ and anti-CD16 antibodies. (**A**) Elisa quantification of IL-10 and TGFβ in CLL-cm from 12 patients revealed the presence of moderate levels of IL-10 (mean: 20 pg/mL) and more consistent concentration of TGFβ (mean: 309 pg/mL). (**B**) The up-regulation of CD16 expression in purified monocytes due to CLL-cm treatment or to TGFβ stimulation was counteracted by the addition of the anti-TGFβ antibody. Results from two assessed CLL cases (CLL N.25, CLL N.37) are depicted. (**C**) Histograms display the reduction in classical osteoclastogenic differentiation (MCSF + RANKL) in monocytes pre-stimulated with CLL-cm in presence of anti-CD16 or -TGFβ antibodies. Statistical significance was determined by using ANOVA and Tukey’s post hoc test. (**D**) Monocytes pre-stimulated with CLL-cm in the presence of an anti-CD16 or -TGFβ antibodies gave rise to a lower number of multinucleated TRAP+ osteoclasts (arrows), as depicted in images from a representative experiment (CLL-cm N.37). Presented images were acquired at the same enlargement. Bars correspond to 25 µm.

Since MCSF + RANKL activation may induce the release of cytokines, we further addressed here if factors, potentially produced within the microenvironment, contribute to enhance CD16 expression. We confirmed that monocytes treated with IL-10 [31] or with TGFβ up-regulated CD16 (Figure 6A). In addition, we demonstrated that healthy monocytes, pre-activated with either IL-10, TGFβ or CLL-cm, gave rise to a higher proportion of mature osteoclasts, characterized by a larger size (BIG), significantly evident for the aforementioned cytokines and in a trend mode for CLL-cm (Figure 6B,C). The evaluation of IL-10 and TGFβ’s presence, in CLL-cm derived from 12 CLL patients, revealed a moderate amount of IL10 per mL and a more consistent concentration of TGFβ (Figure 7A). Furthermore, the addition of a neutralizing anti-TGFβ antibody to monocytes pre-cultured with CLL-cm down-regulated CD16 expression (Figure 7B) and contemporarily inhibited osteoclast differentiation (Figure 7C). Osteoclastogenesis also appeared inhibited by the addition of an anti-CD16 antibody (Figure 7D). It is of interest to note that Maffei et al. previously reported that monocytes from normal donors, cultured with CLL-cm, released higher amounts of IL-10, IL-8 and IL-6, thus, suggesting that both paracrine (from CLL B cells) and autocrine (from monocytes) IL-10 secretion may contribute in enhancing CD16 expression as well as osteoclastogenesis [32].

### 3.5. Immunodetection of Osteoclasts Expressing CD16 and TRAP in Bone Marrow Biopsies

**Figure 8:**
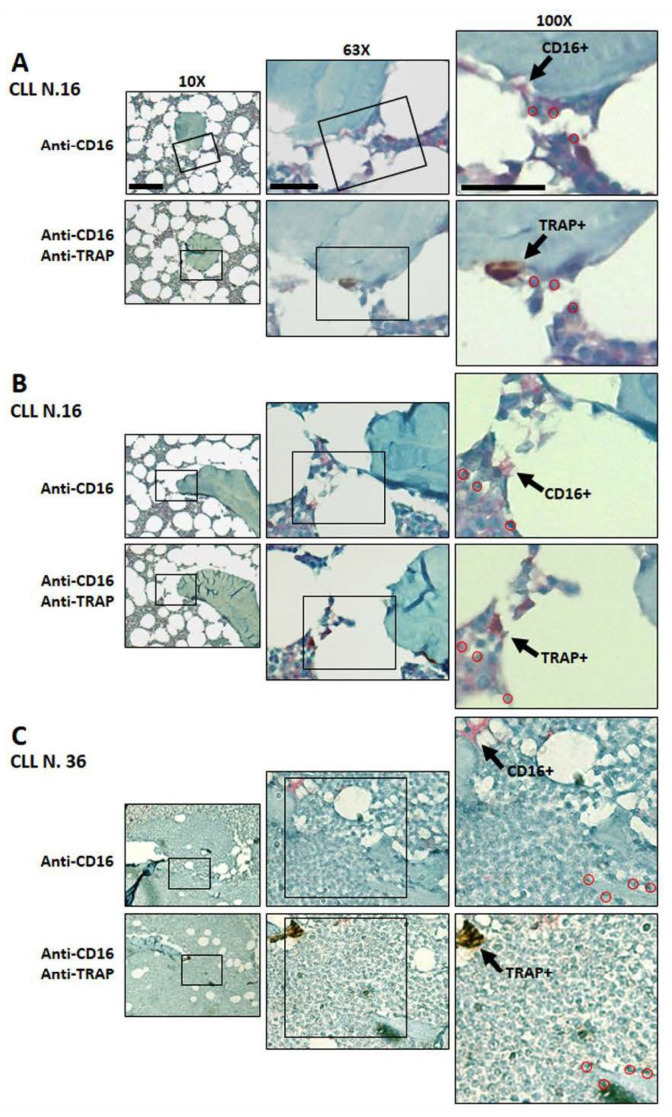
Immunodetection of CD16- and TRAP-positive cells in CLL bone marrow biopsies. (**A**–**C**) Images display progressive enlargements of serial sections to better evaluate CD16 and TRAP positivity in Monocytes/Osteoclasts present in BM biopsies from two CLL patients (N.16, N.36). Single (CD16) or double (CD16/TRAP) positivity could be evidenced in the same cells (arrows). The red circles, identifying cell nuclei positions, were used as references. Bars correspond to 50 µm for 10× images and to 8 µm for 63× and 100× images, respectively.

We then evaluated the potential expression of CD16 in osteoclasts in BM biopsies from two CLL patients (N.16, N.36). To this aim, single and double immune-histochemical staining with specific anti-human CD16 and anti-human TRAP antibodies was performed in serial BM biopsy sections. A high number of CD16-positive cells, diffused within the bone marrow, was detectable in the two BM biopsies (personal observation). As displayed in Figure 8, we further observed that a few TRAP+ cells, located on the bone surface rim, also appeared weakly positive for CD16. This finding parallels the experimental results shown above, thus, suggesting that CD16 appears highly expressed in monocytes and in osteoclast precursors and is further down-modulated, but still present, along their maturation.

### 3.6. CLL-Conditioned Media Stimulate Proliferation and Migration of Healthy Monocytes

**Figure 9:**
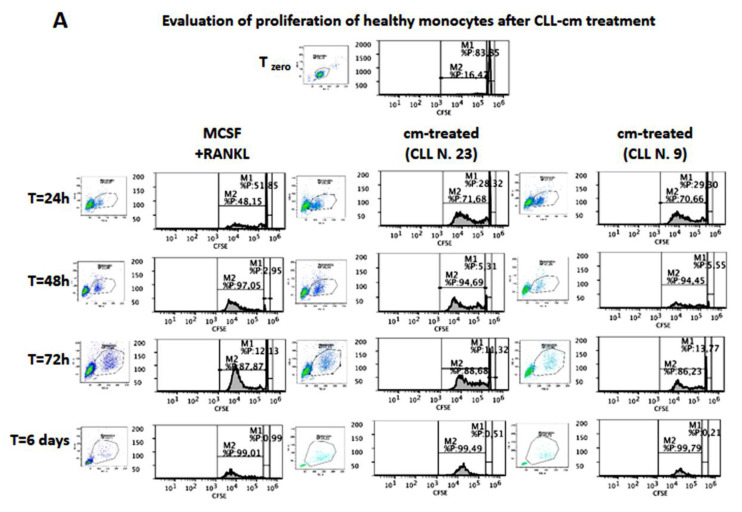

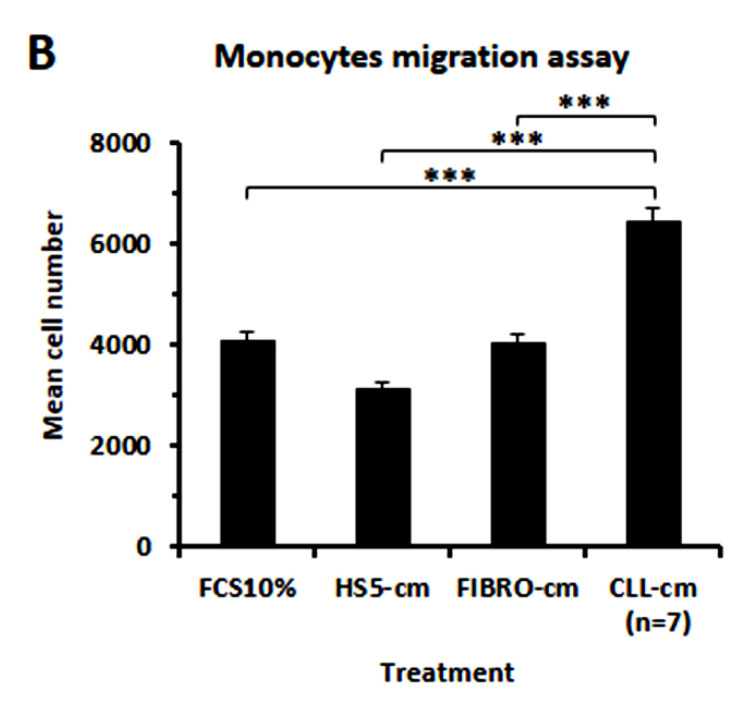
Evaluation of proliferation and migration of healthy monocytes in response to MCSF + RANKL and to CLL-cm treatments. (**A**) CLL-cm treatments stimulated monocytes to gradually divide and proliferate, as evaluated by CFSE staining. CFSE staining at time 0 was evaluated on the total gated population of purified monocytes. In the remaining experimental time course (24 h–6 days) only activated and large monocytes were gated and evaluated to better address mitotic peaks. Results from two representative cases (Cases N. 23 and N. 9) are here depicted. (**B**) CLL-cm treatment further induced a strong and significant migration of healthy monocytes as compared with conditioned medium derived from a stromal (HS5) or a fibroblastic (Fibrogem) cell line or with medium alone (FCS10%). Bars are representative of the mean ± SD of two separate experiments. Statistical significance was determined by using ANOVA and Tukey’s post hoc test.

MCSF and RANKL are commonly utilized to induce osteoclast differentiation from monocytes in vitro: while MCSF induces survival, RANKL stimulates the differentiation. However, it has also been suggested that MCSF triggers proliferation of monocytes and that, following MCSF activation, a higher number of osteoclasts originate from a limited number of proliferating monocytes [33]. We evaluated here whether the proliferation of healthy monocytes can be induced by CLL-cm treatment, as quantified by the CFSE tracer. As shown in Figure 9A, by gating only the activated monocytes, we observed that these cells were gradually induced to divide when cultured with CLL-cm, although with an apparently slower rate in comparison to MCSF + RANKL-stimulated ones.

We further assessed whether healthy monocytes acquire a more striking ability to migrate after culture with CLL-cm: the levels of migration were more robust after CLL-cm treatment than with medium alone (10%FCS) or with medium derived from a stromal and from a fibroblastic cell line (HS5 and Fibrogem, respectively, Figure 9B).

### 3.7. Increased Osteo-Clastogenesis by M2-Polarized Monocytes

**Figure 10:**
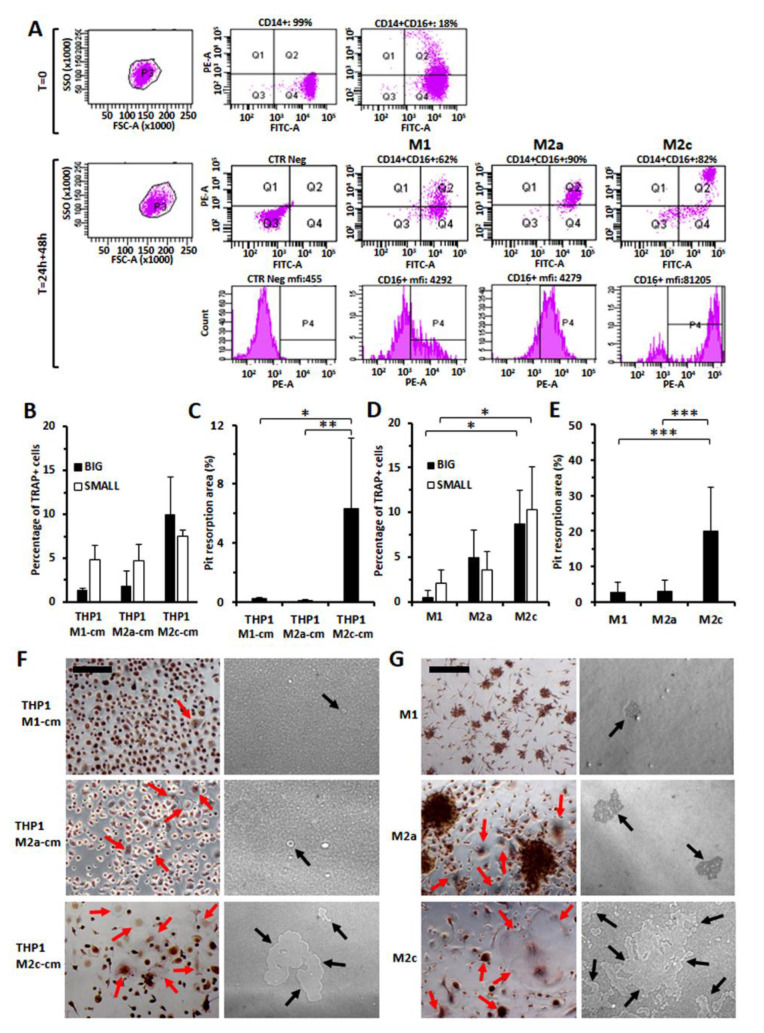
Assessment of CD16 up-regulation in monocytes purified from healthy donors polarized toward M1, M2a and M2c and determination of MCSF + RANKL-osteoclastic differentiation of monocytes pre-treated with culture medium from M1-, M2a-, M2c-polarized THP1 cells or of monocytes directly pre-polarized toward the three subtypes. (**A**) Dot plots show the increased percentage of CD16+ cells when monocytes were polarized toward M2. A very strong CD16 up-regulation was in particular observed in M2c-polarized monocytes (CD16 mfi = 81,205). (**B**) Healthy monocytes pre-treated with culture medium from M2c-THP-1 cells produced a higher number of frank osteoclasts (TRAP+, at least tri-nucleated, of big and small size). Histograms represent means ± SD of two separate experiments using monocytes purified from two different healthy donors. (**C**) Larger pit resorption areas were also detected when healthy monocytes were pre-treated with THP1-M2c-conditioned medium (THP1-M2c-cm), in agreement with the higher number of frank osteoclasts derived. (**D**) M2-polarized monocytes gave rise to a higher number of osteoclasts (means ± SD of two separate experiments), and significant larger areas of erosion were visualized when osteoclasts were derived from M2c pre-polarized cells, as shown in (**E**). Statistical significance was determined by using ANOVA and Tukey’s post hoc test for (**C**–**F**). Osteoclasts originated only from THP1-M2 pre-polarized monocytes and show a larger size for THP1-M2c. (**G**) Images display cell morphological differences acquired after polarization toward M1, M2a and M2c phenotypes and the presence of classical osteoclasts only in M2 pre-polarized cells, showing, in particular, a larger size in M2c. Larger pit resorption areas were detected in M2c-derived osteoclasts. For all images in panels (**F**,**G**) correspond to 25 µm.

We next investigated whether monocyte polarization could enhance CD16 expression and, in parallel, affect osteoclast differentiation. As shown in Figure 10A, monocytes polarized toward the M2 phenotype exhibit higher CD16 expression. Moreover, when healthy monocytes were pre-treated with conditioned media derived from the cultures of the THP-1 monocytic cell line previously polarized toward M1 (LPS+ IFNγ), M2a (IL4) or M2c (IL10), before the addition of MCSF and RANKL, we could observe a more consistent number of osteoclasts for monocytes pre-treated with conditioned media derived from THP1-M2, particularly for THP-1-M2c (Figure 10B,F). In agreement, we observed a larger area of bone resorption (Figure 10C,F). The direct pre-treatment of purified monocytes with PMA+ IL10, to induce M2c polarization, also gave rise to a higher number of osteoclasts (Figure 10D,G) that produced significantly larger areas of bone erosion (Figure 10E,G).

### 3.8. GEP Analysis

**Figure 11:**
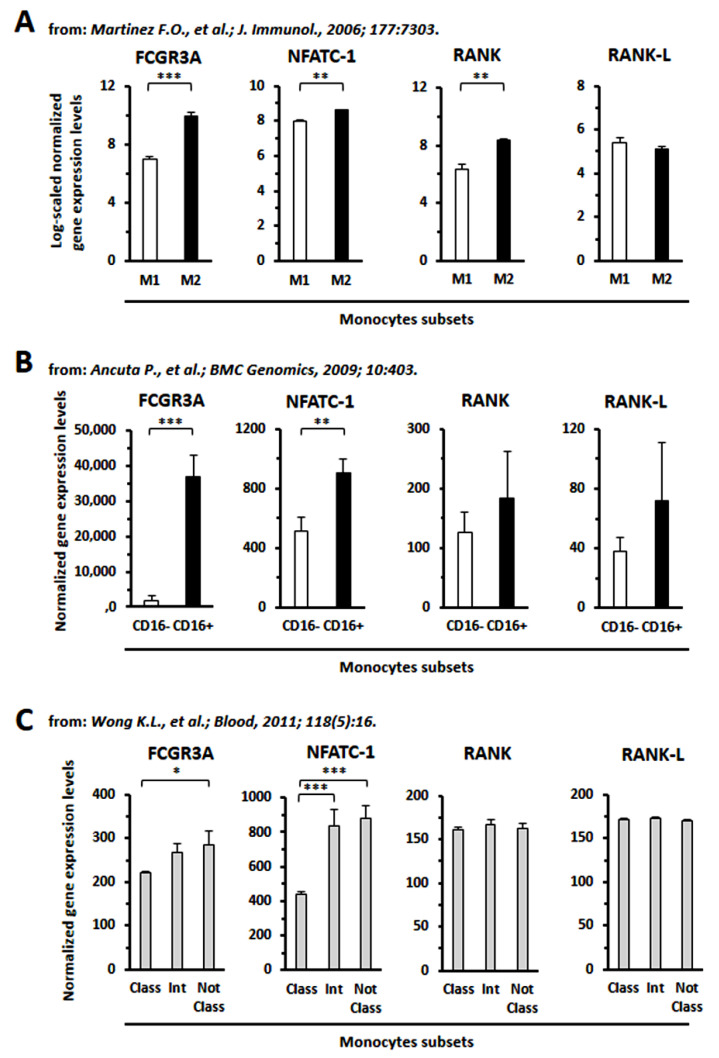
Analysis of gene expression profiles of transcripts for molecules specifically involved in osteoclasto-genesis, as derived from available public literature data. (**A**) Monocytes of type M2 expressed significantly higher transcript levels of CD16 (FCG3A), NFATC1 and RANK in comparison with M1 [24]. (**B**) The expression of NFATC1, the key osteoclastogenic transcription factor, also appeared significantly higher in CD16-positive monocytes versus CD16-negative [26]. (**C**) The “intermediate” and “non-classical” monocytes showed significantly higher levels of NFATC1 in comparison with the “classical subtype” [27]. Statistical significance was achieved with Student *t* test for panels (**A**,**B**) and with ANOVA and Tukey’s post hoc test.

Through the analysis of public gene expression profile data (GSE5099, GSE16836, GSE25139), we could determine that CD16 (FCG3A), NFATC1 and RANK transcript expression was significantly higher in monocytes polarized toward the M2 phenotype versus the M1 phenotype [24] (Figure 11). Higher CD16 expression also appeared related to higher levels of expression of NFATC1, as demonstrated for CD16+ versus CD16- monocytes [26] or for “intermediate” and “non-classical” versus “classical monocytes” [27] (Figure 10). Collectively, these data may further support the experimental results presented above.

## 4. Discussion 

Monocytes/Macrophages are a phenotypically and functionally heterogeneous group of myeloid cells, characterized by a high degree of plasticity. Cytokines produced within the microenvironment strongly affect the acquisition of distinct features on these cells and the abnormal cytokine milieu of the tumor microenvironment may change their phenotype. In general, tumors induce immunosuppression, therefore, recruiting and expanding tumor-associated macrophages (TAMs), which facilitate escaping from the immune control. Monocytes, in CLL patients, are skewed toward an M2 phenotype [1] and also the Nurse-like cells (NLCs), monocytoid cells typically found in this disease [34,35], exhibit immunosuppressive features [1,36]. Indeed, CLL cells shape their microenvironment through direct immunomodulation of accessory cell features [37,38,39]. We have shown that monocytes from normal donors, cultured in the presence of a CLL-conditioned medium, upregulated Indoleamin-2,3 dioxygenase (IDO) and that IDO expression is significantly higher in monocytes from CLL patients than in monocytes from normal controls [1]. In addition, we recently reported that leukemic CLL cells also affect the differentiation of the two major cellular components of the bone tissue, osteoblasts and osteoclasts, leading to deregulation of bone remodeling [4].

CD16 has been proposed as an osteoclast progenitor marker that facilitates osteoclast differentiation [9], although this suggestion is still controversial [40]. We, here, demonstrated that the percentage of circulating monocytes expressing CD16 is significantly higher in Chronic Lymphocytic Leukemia patients than in MBL patients or in normal donors and we further determined that there is a direct correlation between the percentage of “intermediate monocytes” and the entity of bone erosion, in a group of 17 CLL patients evaluated by X-ray CT scan. Higher numbers of monocytes expressing CD16 in CLL patients than in normal donors were also described by Maffei et al. and by Kowalska et al. [32,41]. We also demonstrated here that monocytes from healthy donors, cultured with CLL-cm, strongly up-regulated CD16, and to a lesser extent, RANK and RANKL, further underlining how leukemic B cells influence the phenotype and function of accessory cells in the microenvironment. In addition, we observed that the classical MCSF + RANKL activation, usually employed to generate osteoclasts from monocytes in vitro, or the previous stimulation with IL-10 or TGFβ, induced a significant CD16 up-regulation in healthy monocytes. The enhancement in CD16 in healthy monocytes, detected after IL-10, TGFβ or CLL-cm, appeared paralleled by the increased formation of BIG multinucleated osteoclasts also. These results may collectively indicate that the release of IL10 and of TGFβ by leukemic B cells, stromal cells or by monocytes skewed toward the M2 phenotype, may contribute to increased osteoclasto-genesis. It is of interest to note that healthy monocytes were stimulated to release IL-10, IL-8 and IL-6 when cultured with CLL-cm, as previously reported by Maffei et al. [32]. Moreover, a higher number of osteoclasts was generated when healthy monocytes were pre-treated with supernatants derived from THP1-M2-polarized cells or when directly derived from monocytes activated toward the M2 phenotype, in particular toward M2c. In agreement with this observation, Jihyun Yang et al. demonstrated that M2 monocytes differentiated into osteoclasts more efficiently than M1, especially when pre-activated with IL-10 [42]. These authors highlighted the critical role played by IRF5 in osteoclast differentiation: IRF5 overexpression in M2 monocytes indeed decreased their osteoclastogenic potential, whereas their down-regulation by IRF5 silencing enhanced the osteoclastogenic potential of M1 cells [42]. Interestingly, Kowalska et al. recently showed that the “intermediate” monocytes (CD14++CD16+) are the main producer of IL-10 [43]. This finding may appear in line with our in vivo and in vitro data and may support the hypothesis of a potential relationship among a higher number of “intermediate monocytes”, higher levels of bone erosion found in these patients and the detection of enhanced osteo-clastogenesis by IL-10-driven monocytes. Additional efforts are, nonetheless, needed to clarify the involvement of this subset in bone homeostasis in CLL patients.

It is also of note that, in a particular context, Myeloid-Derived Suppressor Cells (MDSCs) may differentiate towards osteoclast precursors [44,45]. In breast cancer, as reported by Sawant et al., MDSCs localized at bone metastatic sites, differentiated towards osteoclasts and contributed to bone destruction. These authors further outlined the pivotal role played by the tumor microenvironment of the bone in driving MDSC differentiation toward osteoclasts [46,47]. Accordingly, Danilin et al. demonstrated that, in a model of athymic nude mice, MDSCs, after the acquisition of a “tumorigenic phenotype”, promoted breast-cancer-associated bone resorption [48]. In addition, MDSCs, isolated from collagen-induced arthritis (CIA) mice, expressed osteoclast markers and acquired osteoclast bone resorption function after the activation with MCSF + RANKL in vitro [49]. Zhuang J. and collaborators further described that Multiple Myeloma MDSCs, first expanded under the influence of malignant plasma cells, gained the capability of differentiating toward osteoclasts in vitro and in vivo [50]. Collectively, these findings appear intriguing, in the context of the experimental observations here described: the number of MDSCs is, in fact, significantly high in CLL patients and correlates with unfavorable prognostic markers, shorter survival times and overexpression of IDO, ARG1, NOS2, IL-10 and TGFβ [41,51,52]. We, therefore, here hypothesize that the expansion of M2 cells or of MDSCs might, directly or indirectly, participate in increasing the rate of bone tissue resorption observed in these patients.

## 5. Conclusions

**Figure 12:**
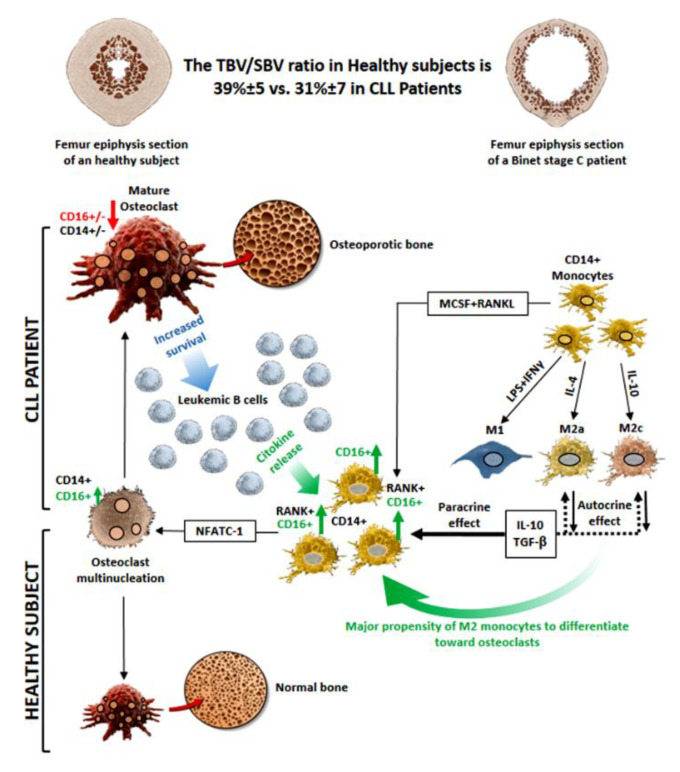
Osteoclast differentiation in CLL patients. Schematic representation of the multiple interactions taking place among leukemic B cells and monocytes leading to an increase in osteoclast maturation and bone resorption in CLL patients. The effects of specific cytokines released in the microenvironment leading to monocyte polarization and/or to CD16 modulation are indicated.

Overall, the present data add new insights to the knowledge of the neoplastic/bystander cell crosstalk, further suggesting that, in CLL patients, the multiple mutual interactions taking place between leukemic B cells and accessory cells in the microenvironment interfere with the physiological bone homeostasis and create a self-sustaining mechanism of activation, survival and expansion of the neoplastic B cell clone, as schematically represented in Figure 12. 

## Data Availability

The data presented in the present manuscript are available on request from the corresponding author.

## Supplementary Materials

The following supporting information can be downloaded at: https://www.mdpi.com/article/10.3390/cancers14235979/s1, Table S1: Main patients’ characteristics (Stage, IgHV, CD38 expression).
