# Supplementary material for: A High Percentage of CD16+ Monocytes Correlates with the Extent of Bone Erosion in Chronic Lymphocytic Leukemia Patients: The Impact of Leukemic B Cells in Monocyte Differentiation and Osteoclast Maturation
Source: Cancers (Basel). 2022 Dec 3;14(23):5979. doi: 10.3390/cancers14235979 (PMC9740193; doi:10.3390/cancers14235979)
Supplement: Supplementary file 1 [file cancers-14-05979-s001.zip › cancers-2056960-supplementary.pdf]

Supplementary Table S1

CASE N.	STAGE (BINET)	IgHV	CD38
N.1	A	MUT	neg
N.2	A	MUT	neg
N.3	B	MUT	neg
N.4	C	UNMUT	pos
N.5	B	MUT	pos
N.6	A	MUT	pos
N.7	A	MUT	neg
N.8	A	UNMUT	nd
N.9	A	UNMUT	nd
N.10	A	UNMUT	nd
N.11	A	UNMUT	pos
N.12	A	UNMUT	pos
N.13	B	MUT	neg
N.14	B	MUT	pos
N.15	B	MUT	neg
N.16	B	UNMUT	pos
N.17	C	MUT	neg
N.18	C	UNMUT	neg
N.19	C	MUT	pos
N.20	C	MUT	neg
N.21	B	UNMUT	neg
N.22	A	UNMUT	nd
N.23	C	UNMUT	nd
N.24	B	UNMUT	neg
N.25	A	UNMUT	pos
N.26	A	MUT	neg
N.27	B	MUT	pos
N.28	A	MUT	pos
N.29	nd	MUT	pos
N.30	A	UNMUT	pos
N.31	A	MUT	pos
N.32	A	UNMUT	nd
N.33	A	MUT	pos
N.34	A	UNMUT	pos
N.35	C	UNMUT	pos
N.36	C	UNMUT	nd
N.37	C	MUT	pos
